# Alteplase associated Orolingual angioedema: A case report and literature review

**DOI:** 10.1097/MD.0000000000032474

**Published:** 2022-12-30

**Authors:** Xiuyan Qi, Huiqian Lin

**Affiliations:** a Department of Neurology, Shijiazhuang People’s Hospital, Hebei, China.

**Keywords:** ACEI, acute ischemic stroke, intravenous thrombolysis, orolingual angioedema, recombinant tissue plasminogen activator

## Abstract

Orolingual angioedema (OA) is a rare but life-threatening complication of intravenous thrombolysis using alteplase. Angioedema can be caused by almost any medication. Administration of recombinant tissue plasminogen activator causes atypical angioedema. This study aimed to investigate factors related to and treatment of OA after thrombolysis with alteplase.

**Case report:**

We describe the case of a 53-year-old man with a history of hypertension managed with enalapril, who presented with ischemic cerebrovascular stroke. Intravenous alteplase was administered, and within 54 minutes, the patient developed severe orolingual edema requiring emergent intubation. Subsequent imaging revealed an acute-to-subacute infarct in the left occipital lobe of the posterior cerebral artery.

**Results:**

The most common factor for increased risk of OA after recombinant tissue plasminogen activator was concomitant use of angiotensin-converting enzyme inhibitors (ACEI).

**Conclusion:**

Before intravenous thrombolytic therapy, patients should be asked if they have a history of allergies, are currently using ACEI, and try to avoid using ACEI antihypertensive drugs before and after thrombolytic therapy.

## 1. Introduction

Intravenous injection of recombinant tissue plasminogen activator (RT-PA) is an effective method for the treatment of acute ischemic stroke and can improve the survival rate and reduce the mortality rate of patients with acute ischemic stroke.^[1]^ However, in addition to intracranial hemorrhage, Orolingual angioedema (OA) is a rare but potentially life-threatening complication is increasing.^[2]^ OA presents as acute swelling of the tongue, lips, or face and can be life-threatening because it increases the risk of upper airway obstruction.^[3]^ However, the mechanisms underlying RT-PA-induced OA remain unclear. Increasing evidence suggests that RT-PA-induced OA is primarily driven by bradykinin and that close monitoring is important. Several studies have found that the use of angiotensin-converting enzyme (ACE) inhibitors is associated with an increased risk of angioedema following RT-PA.^[4]^ Some studies have also suggested that the female sex and insular infarction may be risk factors for OA. However, sex and infarct site were not found to be directly correlated. Herein, we report a case of a male patient with posterior circulation infarction who developed OA after intravenous thrombolysis (IVT). Previously, the patient had a rash on his chest after drinking alcohol, and he was usually administered enalapril to reduce blood pressure. Therefore, close observation during and after alteplase treatment is recommended.

## 2. Case report

**Figure 1.**
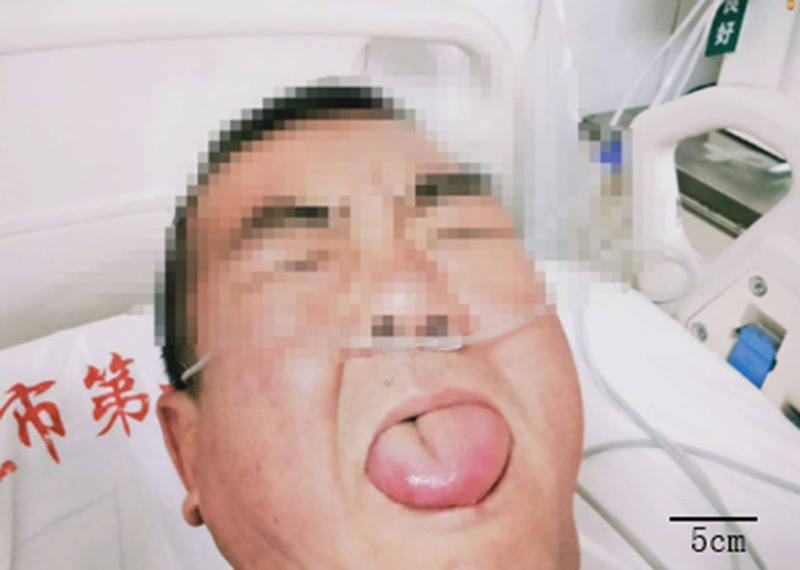
Orolingual angioedema after tissue plasminogen activator infusion.

**Figure 2.**
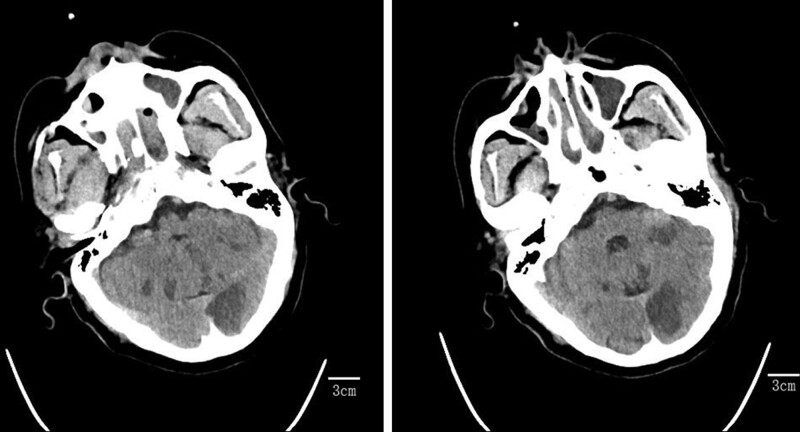
Head CT reexamined 24 hours after intravenous thrombolysis showed posterior circulation cerebral infarction. CT = computed tomography.

A 53-year-old male who presented to the emergency department with a stroke call due to dizziness, left limb weakness, and slurred speech. Upon arrival, the patient was brought directly to the computed tomography (CT) for imaging, where the neurology stroke team awaited assessment. On presentation, the patient’s glucose level was normal. A review of patients prior to admission showed that they were taking enalapril 10 mg QD. Past allergies, as evidenced by a rash on the chest after drinking. Physical examination revealed clear consciousness, dysarthria, shallow left nasolabial groove, skewed mouth angle to the right, tongue extension to the left, left limb muscle strength grade 0, low muscle tension, and a left Babinski sign (+). The National Institutes of Health Stroke Scale (NIHSS) score was 12; 2 points for dysarthria, 2 points for partial facial paralysis, and 8 points for left limb weakness. No hemorrhage or new infarction was found on the head CT scan. The patients were evaluated and given intravenous thrombolytic therapy with alteplase at the international standard dose of 0.9 mg/Kg after meeting the indications of RT-PA. The patient weighed 75 kg and the total amount of alteplase was 67.5 mg. Alteplase 6.75 mg (10% of the total) was injected intravenously and the remaining 50.75 mg (90% of the total) was pumped intravenously for 1 hour. During medication observation, the patient complained that the dizziness symptoms were better than before, speech was clearer than before, left limb activity was normal, NIHSS score was 2, and left tongue swelling occurred 54 minutes after thrombolysis (Fig. 1). The alteplase pump was immediately stopped and methylprednisolone and diphenhydramine were administered. There was no improvement after treatment, and the patient gradually appeared on the right side of the tongue swelling, administered dexamethasone atomization inhalation and a higher oxygen flow of 8 L/min. The patient developed laryngeal distension and was administered an oropharyngeal tube with unobstructed airway, subcutaneous adrenaline injection, budesonide aerosol inhalation, and calcium gluconate. The patient was then transferred to the intensive care unit for further rescue. 24 hours later, the patient’s symptoms had completely resolved. The symptoms did not recur during hospitalization. The patient’s head CT reexamination 24 hours after thrombolysis showed a posterior circulation cerebral infarction (Fig. 2). After 14 days of hospitalization, his symptoms improved and he was discharged.

## 3. Discussion

Acute oropharyngeal angioedema associated with IVT with alteplase is a potentially life-threatening complication in patients with acute ischemic stroke, with a frequency of 0.2% to 7.9%.^[5]^ Angioedema is an acute and self-limiting swelling of subcutaneous or submucosal tissues due to increased vascular permeability resulting from the extravasation of fluid from the loss of vascular integrity. Alteplase is a recombinant serine protease that cleaves plasminogen to plasmin, which can activate the complement and kinin pathways in addition to its thrombolytic effects. Alteplase induces the release of bradykinin in plasma in vitro by activating plasmin.^[6]^ As ACE is also involved in bradykinin degradation, angiotensin-converting enzyme inhibitors (ACEI) may lead to increased serum concentrations of these inflammatory mediators. ACE inhibitors are the most recognized predisposing factors for OA.^[7]^ One hypothesis is a bradykinin-mediated pathway in which RT-PA hydrolyzes plasminogen to plasmin, which in turn activates the kinin pathway and increases bradykinin production.^[8]^ Bradykinin is a potent pro-inflammatory and pro-edematous peptide that increases vascular permeability and vasodilatation, leading to angioedema. Additionally, RT-PA can increase histamine levels and cause vasodilatation.^[9]^ Other hypotheses suggest that RT-PA and plasmin activate the complement pathway and increase complement levels, which in turn activate the degranulation of mast cells, histamine, and basophils, leading to angioedema.^[9]^ In patients taking ACE inhibitors, the risk of angioedema after thrombolysis increases because these agents increase bradykinin concentration.^[10]^ In addition, neurokinins such as substance P are mediators of inflammation and angioedema.^[11]^ Elevated RT-PA-associated bradykinin levels, decreased ACEI-mediated bradykinin metabolism, and elevated neurokinin levels associated with ACE inhibitors have been combined to increase the risk of angioedema.

OA is a rare complication of RT-PA IVT. In severe cases, the patient’s life may be endangered. It is essential to stop RT-PA perfusion when signs of angioedema are present. Most patients require a combination of supportive medications such as corticosteroids, antihistamines, and epinephrine to treat angioedema. Severe oropharyngeal angioedema necessitates intubation to protect the airways. Symptom relief ranged from shortly after dosing to 72 hours after symptom onset. OA after RT-PA administration can cause serious complications, suggesting that patients should be closely monitored for several hours to prevent airway injuries. The risk of OA after intravenous thrombolytic therapy with RT-PA increases if the patient takes ACEI drugs before OA onset. ACEI should not be the first-choice antihypertensive agent for patients who require blood pressure reduction before administration. The prevalence of OA should be monitored during RT-PA intravenous thrombolytic therapy. Timely detection and early intervention can prevent this disease effectively.

## 4. Conclusion

OA caused by RT-PA is rare but life-threatening. The risk was increased in patients taking angiotensin-converting enzyme inhibitors. Although evidence suggests varying degrees of improvement with antihistamines, steroids, epinephrine, and complement inhibitors, OA is typically self-limiting. Owing to the severity of oropharyngeal angioedema and the progression of airway damage, close monitoring is critical. Endotracheal intubation should be performed if necessary. Avoiding tracheotomy.

## Author contributions

**Writing – original draft:** Xiuyan Qi.

**Writing – review & editing:** Huiqian Lin.
